# Effect of Grape Pomace Addition on the Technological, Sensory, and Nutritional Properties of Durum Wheat Pasta

**DOI:** 10.3390/foods9030354

**Published:** 2020-03-19

**Authors:** Roberta Tolve, Gabriella Pasini, Fabiola Vignale, Fabio Favati, Barbara Simonato

**Affiliations:** 1 Department of Biotechnology, University of Verona, Strada Le Grazie 15, 37134 Verona, Italy; roberta.tolve@univr.it (R.T.); fabiola.vignale@gmail.com (F.V.); fabio.favati@univr.it (F.F.); 2 Department of Agronomy, Food, Natural Resources, Animals and Environment, University of Padova, Viale dell’Università 16, 35020 Legnaro Padova, Italy; gabriella.pasini@unipd.it

**Keywords:** agro-industrial by-product, fortified pasta, dietary fiber, phenolic compounds, starch digestibility

## Abstract

In this study, fortified pasta was prepared by replacing semolina with 0, 5, and 10 g/100 g of grape pomace (GP), a food industry by-product, rich in fiber and phenols. GP inclusion in pasta significantly reduced its optimum cooking time and the swelling index, while also increasing the cooking loss (*p* < 0.05). Furthermore, pasta firmness and adhesiveness were enhanced by the GP addition, as well as the total phenol content and the antioxidant activity, evaluated through ABTS and FRAP assays (*p* < 0.05). From a nutritional point of view, increasing amounts of GP resulted in a significative decrease in the rapidly digestible starch and an increase in the slowly digestible starch, while the predicted in vitro glycemic index was also reduced (*p* < 0.05). Sensory analysis showed that fortified spaghetti had good overall acceptability, and the results suggest that GP-fortified pasta could represent a healthy product with good technological and sensory properties.

## 1. Introduction

Pasta, a staple food consumed worldwide, can represent an excellent choice for the addition of bioactive compounds [1,2]. Considering the concepts associated with the circular economy, the possibility of using food industry by-products as a source of bioactive compounds, such as antioxidants and dietary fibers [3,4], is of interest. Among the various food industry by-products with potential healthy properties, grape pomace (GP), a residue of grape processing in wine production, may be an interesting ingredient. GP represents about 20% of the mass of total processed grapes and it is estimated that for every 100 liters of produced wine, about 17 kg of GP must be disposed. Grape skins represent more than 80% of the wet weight of GP and being rich in phenolic compounds and dietary fiber may be an interesting and cheap source of healthy moieties [5,6,7]. In humans, phenolic compounds exert a wide range of beneficial physiological activities, and many epidemiological studies associate a phenolic-rich diet with the prevention of several pathologies, such as cardiovascular diseases, diabetes, as well as some types of cancer [8,9,10]. GP also contains a noticeable quantity of dietary fiber (DF), whose level depends on several factors, among which is the grape variety. For red grapes, the DF content has been reported to range between 51% and 74% (by weight on dry matter) and the DF consumption may help in reducing the incidence of some types of cancer, as well as the development of diabetes. Moreover, fibers improve satiety and intestinal peristalsis, favor blood cholesterol decrease, and prevent obesity [11,12,13,14]. While the recommended DF intake is 25–30 g per day, this value is often not reached because of the modern eating habits, and the availability of fiber-enriched or fortified foods may represent a good opportunity for consumers to increase their daily fiber intake [15].

Nowadays, pasta is a product consumed worldwide and represents a remarkable staple food to convey bioactive compounds. To achieve this, several studies have been carried out to develop pasta with enhanced nutritional properties by using, for instance, olive pomace, carrot pomace, and tomato. The addition of these ingredients in pasta formulation, as well as the increase in dietary fiber and antioxidant activity, generally result in the modification of the technological and cooking properties, starch digestibility, and glycemic index [4,16,17,18]. In this framework, GP can represent a valuable ingredient to produce fortified food items, and this study aimed to evaluate the effects of replacing durum wheat semolina with different levels of GP in the production of durum wheat spaghetti (0/100, 5/95, and 10/90 g GP/g semolina). The effects on pasta quality were assessed for cooking properties, color, and texture. The nutritional properties of GP-fortified pasta were also evaluated, considering the total polyphenol content, the antioxidant capacity and the “in vitro” starch digestibility. Finally, the sensory properties of the fortified prepared spaghetti were also appraised.

## 2. Materials and Methods

### 2.1. Grape Pomace Powder Preparation and Chemical Composition

Grape pomace from *Vitis vinfera* L cv. Corvina, a red grape used for Amarone wine production, was kindly supplied by Tinazzi srl (Verona, Italy). The GP, after alcoholic fermentation, was separated, pressed, and immediately recovered and dried in a vacuum oven (VD 115 Binder GmbH, Tuttlingen, Germany) (40 °C, 30 kPa) until reaching a final moisture content of 11.0 g water/100 g GP. Afterwards, the stems and seeds were manually removed from the pomace and the by-product was milled (GM200 Retsch, Haan, Germany) to obtain a powder with a particle size < 0.2 mm. The GP was then stored in an airtight, dark plastic container until analyzed or used for pasta preparation. The GP chemical composition was assessed in triplicate according to the following AOAC standard methods: 930.15 for moisture, 976.05 for protein, 985.29 for total fiber, and 942.05 for ash content [19]. All chemical components were expressed as g/100 g of dry matter (DM).

### 2.2. Pasta Preparation

Commercial durum wheat semolina was purchased in a local market, and the following is the nutrient composition reported on the label: carbohydrates 71.8 g/100 g, protein 11 g/100 g, fat 1.8 g/100 g, fiber 3 g/100 g. Pasta was prepared, replacing semolina with different GP amounts, obtaining the samples GP0, GP5, and GP10 (0/100, 5/95 and 10/90 g GP/g semolina). The dough was prepared using a professional pasta machine (Mod. Lillodue, Bottene, Marano Vicentino, Italy) by adding 35% of tap water at 40 °C to pure semolina or to the blend GP-semolina. The dough was mixed for 10 min before being extruded through a 1.75-mm bronze spaghetti die, cutting the spaghetti at the standard industrial length of 250 mm. The spaghetti were air-dried at 50 °C in a Juan laboratory drier (Thermo Fisher Scientific, Waltham, MA, USA) until reaching a residual moisture content of 12 g water/100 g pasta. The pasta samples were then cooked in boiling distilled water in a ratio 1:10 (w/v). The optimum cooking time was assessed experimentally.

### 2.3. Pasta Properties Determination

Pasta moisture content, optimum cooking time (OCT), and cooking loss (CL) were assessed according to the AACC methods 44-15A and 66-50 [20]. The swelling index (SI) was measured according to the procedure reported by Clearly and Brennan [21].

### 2.4. Color Analysis

Uncooked and cooked spaghetti color was measured with a reflectance colorimeter (Minolta Chroma meter CR-300, Osaka, Japan) (illuminant D65) following the CIE - L* a* b* color system. Each measure was made in triplicate.

### 2.5. Texture Analysis

A TA-XT plus Texture Analyser (Stable Micro Systems, Godalming, UK) equipped with a 5-kg load cell was used to measure the cooked pasta firmness and adhesiveness. The firmness test was performed according to the AACC 16-50 method [20]. Samples were put side by side on the lower plate perpendicularly to a probe and compressed by a superior plate (speed 2 mm/s, percentage of deformation 75%). The firmness was recorded as the maximum force required to compress the pasta samples, and the adhesiveness was defined as the negative peak force required to separate the probe from the sample surface.

### 2.6. Determination of Total Phenolic Compounds, ABTS, and FRAP Assay

One gram of each sample (GP, GP0, GP5, GP10) was extracted for 24 h, at room temperature, with 15 mL of MeOH:HCl (97:3) under continuous stirring in the dark [22]. After centrifugation at 3500 g for 10 min at 4 °C, the supernatant was recovered and utilized for assessing the total phenolic content (TPC), ABTS and FRAP radical scavenging activities. The TPC was determined according to Singleton and Rossi [23] with slight modifications. In detail, 0.2 mL of the extract was mixed with 0.2 mL of Folin-Ciocalteau reagent. After 5 min, 4 mL of Na2CO3 solution (0.7 M) was added and the final volume was brought to 10 mL using Milli-Q water. After 1 h, the absorbance of the solution was measured at 725 nm (ATi Unicam UV2, Akribis Scientific, Cambridge, UK). The TPC was expressed as mg of gallic acid equivalent (GAE)/g of dry matter (DM). The ABTS assay was performed according to the method proposed by Del Pino-García et al. [22]. A stock solution of the radical cation (ABTS^•+^) was prepared by incubating in the dark for 12 h, at room temperature, with ABTS (7 mM) and K2S2O8 (2.45 mM) (1:1 ratio). Subsequently, 0.2 mL of the methanolic extract obtained as described were added to 9.8 mL of ABTS^•+^ working solution and incubated at room temperature, in the dark, for 30 min. Absorbance was measured spectrophotometrically at 734 nm. The results were expressed as the µM of Trolox equivalent (TE)/g of DM, using a Trolox calibration curve. The FRAP assay was performed according to Benzie and Strain [24]. The FRAP reagent was obtained by mixing in a volume ratio of 10:1:1, a sodium acetate buffer (300 mM, pH 3.6), TPTZ solution (10 mM) in HCl (40 mM), and a FeCl3.6H2O solution (20 mM). A total of 10 μL of the methanolic extract were mixed with 1 mL of MilliQ water and 1.8 mL of FRAP reagent. The absorbance was then measured at 593 nm and the results expressed as µM of TE/g of DM, using a Trolox calibration curve.

### 2.7. Starch Fractions Determination

Rapidly digestible starch (RDS) and slowly digestible starch (SDS) were measured in cooked pasta samples according to the method of Englyst et al. [25], slightly modified. Briefly, the starch hydrolysis of pasta cooked to the optimum was performed at 37 °C using an enzyme mixture composed by pancreatic amylase (1350 U), amyloglucosydase (3300 U), and invertase (2000 U). The amount of glucose released after pasta starch hydrolysis was measured spectrophotometrically at 510 nm using a glucose oxidase kit (GOPOD, Megazyme, Ireland). The RDS value was calculated considering the amount of glucose released after 20 min of enzymatic reaction. SDS was determined by subtracting the glucose released after 20 min from the glucose released after 120 min. The resistant starch (RS) was measured according to the Megazyme protocol (K-RSTAR, Megazyme, Ireland).

### 2.8. Hydrolysis Index and Predicted Glycemic Index

The hydrolysis index was determined according to Simonato et al. [4]. Briefly, 100 mg of cooked pasta was incubated at 37 °C in glass vials, adding 4 mL of maleic buffer (pH 6) containing 40 mg of pancreatic α-amylase (3000 U/mg) and 4 µL of amyloglucosidase solution (300 U/mL) (Megazyme Ltd.). After 0, 30, 60, 120, and 180 min, the reaction was stopped by adding 4 mL of pure ethanol, and the solution was centrifuged at 2500 g for 10 min. D-Glucose was assessed as described above. The hydrolysis index (HI) was considered as the percentage between the area under the hydrolyses curve (0–180 min) of each pasta sample and the corresponding area of a white bread curve, used as a reference. The predicted glycemic index (pGI) was measured according to the formula proposed by Granfeldt et al. [26]: pGI = 8.198 + 0.862 × HI.

### 2.9. Scanning Electron Microscopy

The microstructure of cross sections of uncooked and cooked pasta samples were observed using a TM-1000 Environmental Electronic Scanning Microscope (Hitachi High-Technology, Tokyo, Japan) equipped with an accessory door tilt and rotate port plate (Deben, UK) at room temperature. The pasta samples were viewed using 1200× magnification, and the images were processed with AdobePhotoshop 6.0.

### 2.10. Sensory Evaluation

The sensory profile of pasta samples was assessed by using a panel of 30 individuals (14 men, 16 women; 23–54 years old) and the informed consent was obtained from each subject prior to their participation in the study. The judges were trained to recognize different intensities of 12 sensory attributes (Appearance: typical semolina pasta color, color uniformity, starch release; Taste: sweet, acid; Aroma: pasta, wine, acid; Texture: astringent, roughness, adhesiveness, graininess). Each judge received 15 g of cooked pasta placed in a container with a lid. The order of sample presentation was balanced and randomized among judges. A 9-point scale, in which 1 represented the lowest intensity and 9 the highest intensity for each attribute, was used. Mean sensory scores from the 30 panel members for each attribute were then calculated. Panelists were asked also to give a comment on the overall acceptability of pasta.

### 2.11. Statistical Analysis

The analyses were carried out in triplicates and the mean values ± standard deviation are reported. The variables were tested for significance using one-way analysis of variance (ANOVA). Differences among means were assessed using Tukey’s HSD test (*p* < 0.05). All the statistical analyses were carried out using the statistical software R project (version 3.2.3 December 2015) [27].

## 3. Results and Discussion

### 3.1. Grape Pomace Composition

The assessed chemical composition of the dried GP was as follows: moisture (11.0 ± 0.2 g/100 g DM), protein (11.19 ± 0.97 g/100 g DM), total fiber (52.3 ± 2.1 g/100 g DM), ash (4.17 ± 0.87 g/100 g DM).

### 3.2. Cooking, Textural Properties, and Color Value

Cooking and textural properties of control and fortified pasta samples are reported in Table 1. GP addition caused a significant reduction in the OCT values (*p* < 0.05), and this could be explained considering that high GP levels may cause a decrease in the overall gluten quantity, affecting the starch–protein structure and, hence, the texture and cooking properties of the spaghetti [28]. The high GP fiber content (52.3 ± 2.1 g/100 g GP), in comparison to the content of the control (3 g/100 g) could contribute to altering the gluten matrix, therefore allowing swift water entry into the pasta central core during cooking and inducing an earlier starch gelatinization and OCT reduction. CL is an index of the capability of the pasta starch–protein matrix to retain its structural integrity during cooking [29].

It has been reported that the addition of fiber can cause a CL increase as fiber may interfere with the starch gluten network, causing its weakening. Consequently, starch gelatinization could occur more rapidly, justifying the OCT reduction and higher leaching of the gelatinized starch from the pasta into the water during cooking [30,31]. The experimental data showed that GP addition caused a significant CL increase, both in GP5 and GP10 spaghetti (*p* < 0.05). Pasta fortification caused a significant reduction of the SI value (*p* < 0.05) that could be ascribed to the competition for water between fiber and starch during pasta cooking. Fiber addition through GP fortification also caused an increase in spaghetti firmness (*p* < 0.05). A similar trend has been reported in the literature when studying the enrichment of durum wheat spaghetti with Barley BalanceTM, even if opposite results have been reported when using different sources of fiber (e.g., inulin, guar gum) [28]. Adhesiveness also showed a significant increment in the GP5 and GP10 samples with respect to plain spaghetti. This could be ascribed to the breaking of the continuous structure of the pasta due to the fiber addition [32].

With regards to the pasta color, Table 2 shows that a progressive increase in GP concentration led to a significant reduction in lightness (L*) both in uncooked and cooked spaghetti. An increase in redness (a*) was also evident along with a GP increase for both uncooked and cooked pasta with a major extent for the latter. Moreover, redness values for uncooked GP5 and GP10 were not significantly different, unlike what was observed in the same samples of cooked pasta. A significant decrease in yellowness (b*) was evident with the progressive addition of GP in both cooked and uncooked spaghetti compared to GP0.

### 3.3. Polyphenols and Antioxidant Activity

The TPC quantity of GP powder was 34.45 ± 0.38 mg GAE/g DM, while the antioxidant activity was 393.43 ± 2.58 and 171.18 ± 0.74 µM TE/g DM evaluated by FRAP and ABTS, respectively. In the fortified spaghetti, the TPC content, as well as the antioxidant activity assessed by ABTS and FRAP assays, showed to be significantly higher in comparison to the control (*p* < 0.05) (Table 3). The TPC and antioxidant activity were positively correlated to the quantity of GP added, both in uncooked and cooked to the optimum spaghetti (r > 0.87). The cooking treatment caused a significant decrease (*p* < 0.05) in the antioxidant activity and of the assessed TPC values, whose reduction ranged from 24 to about 30% in GP5 and GP10 samples. Similar results are reported in the literature and could be ascribed to the degradation of phenolic compounds during cooking or to their leaching into the cooking water [4,17].

However, despite the recorded TPC losses, cooked pasta still has a good antioxidant capacity due to the polyphenol compounds retained in the structure. GP fortification caused a significant increase in the antioxidant activity of the pasta samples, both in the uncooked and cooked states (*p* < 0.05), and the measured antioxidant activity showed to be strictly correlated to the quantity of GP added to the dough according to both ABTS (r = 0.82) or FRAP (r = 0.93) assay (Table 3).

### 3.4. In Vitro Starch Digestibility

The evaluation of the nutritional quality of GP-fortified pasta, in terms of starch digestibility, was carried out using a well-established in vitro assay, validated also in vivo and recognized by the European Food Safety Authority [33]. On the base of the hydrolysis rate and extent, starch can be classified into different fractions: rapidly digestible starch (RDS), slowly digestible starch (SDS), and resistant starch (RS) [25]. The obtained data are reported in Table 4, together with the predicted glycemic index. In comparison to the control, increasing GP amounts in the dough caused a reduction in the RDS value, responsible for a rapid increment of glucose and insulin levels in the blood. The reduction was statistically significant for the GP10 sample, the RDS value being about 13% lower than that assessed in the control (*p* < 0.05). SDS is the starch fraction that undergoes slow digestion, allowing to maintain the glucose level in the blood over time and being a good indicator of the glycemic response in humans [34].

The SDS level was also affected by increasing the amount of GP in spaghetti, reaching a significant increment of about 10% in the GP10 pasta sample (*p* < 0.05). With regards to the RS, the experimental data showed a reduction trend with the use of increasing GP amount in pasta. However, no significant differences could be highlighted among the various samples. The pGI, useful for predicting the likely in vivo glycemic response, showed a significant decrease (*p* < 0.05) with the progressive addition of GP in pasta. Overall the data reported in Table 4 indicate that GP inclusion in pasta positively affected the rate of starch digestibility. The lower glucose release observed in this study could be partially due to the reduction in the starch content in the pasta because of the replacement of semolina with different GP amounts or the starch leaching into the water during cooking, as also reported in the literature [4,30]. It should be pointed out that GP comprises about 52% of fiber that can compete with the starch granules for water adsorption. Therefore, starch gelatinization could be reduced, as well as the action of the starch hydrolyzing enzymes, resulting in a limited starch digestibility [35]. Moreover, GP was rich also in phenolic compounds, such as phenolic acids, flavonoids, and tannins, that can inhibit the α-amylase and α-glucosidase activities, as previously reported by Hanhineva et al. [36]. Hence, the TPC increase in GP pasta could also contribute to the decrease in the rate of starch digestion.

### 3.5. Scanning Electron Microscopy (ESEM)

**Figure 1:**
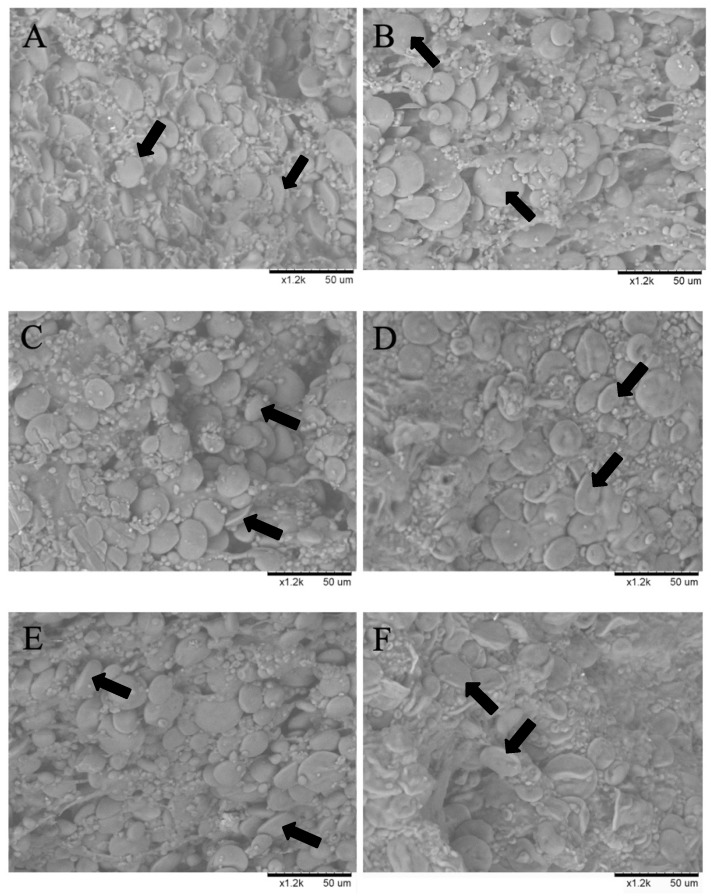
SEM micrographs of uncooked (left) and cooked (right) GP0 (**A**,**B**), GP5 (**C**,**D**), and GP10 (**E**,**F**) pasta samples. The black arrows indicate starch granules.

ESEM observations of cross sections of control (GP0), GP5, and GP10 spaghetti reveal a well-developed protein matrix with circular and lentil-shaped starch granules trapped in the gluten network (Figure 1).

The structural differences observed in raw pasta are more evident after cooking, especially for the GP10 spaghetti sample, where the protein network appears to be less structured. GP0, GP5, and GP10 spaghetti show a similar pattern, in which swollen starch granules are completely embedded in the matrix. However, the images of the cooked fortified samples highlight a lower presence of a well-structured filamentous protein network in comparison with the control.

### 3.6. Sensory Evaluation

**Figure 2:**
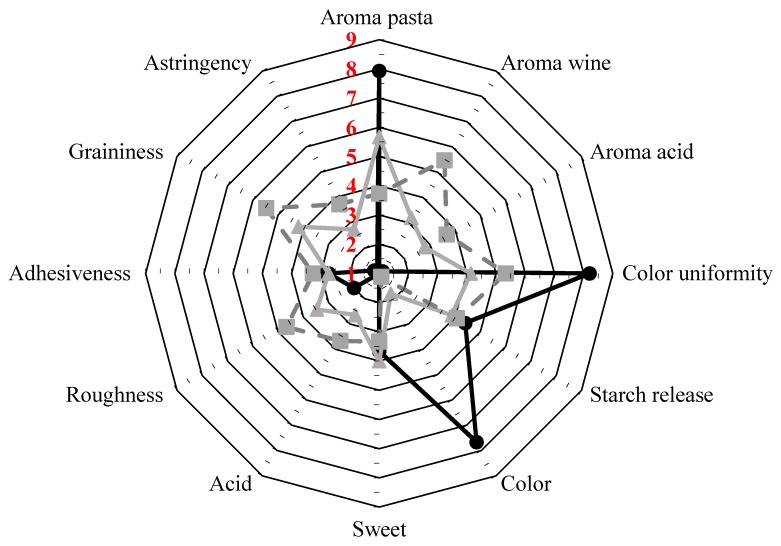
Sensory scores of quality attributes of pasta fortified with grape pomace at different addition levels (GP0 black solid line; GP5 solid gray line; GP10 dashed gray line).

Sensory evaluation of the spaghetti samples revealed that the substitution of semolina wheat with GP powder significantly affected most of the selected attributes (Figure 2).

Using increasing amounts of GP, the perception of the aroma pasta and semolina pasta color decreased (*p* < 0.05). Aroma wine, aroma acid, flavor acid, astringency, and graininess, not or slightly detected in the control sample, were significantly perceived, with the GP10 sample being significantly different from the GP5 one. Roughness was also influenced by the GP addition, and the fortified samples were perceived as being rougher than the control (*p* < 0.05). As demonstrated by the instrumental evaluation of pasta color, the inclusion of GP caused a significant variation that the panelists perceived regardless of the GP concentration used. As far as color uniformity, GP addition caused its reduction (*p* < 0.05) and the lowest score was recorded for the GP5 sample, where the amount of added GP could not uniformly hinder the native semolina color. For starch release, adhesiveness, and sweet the use of GP did not cause any statistical difference. However, a slight adhesiveness increase in GP-fortified spaghetti was assessed by the panel, even if not significant. This is somehow in contrast with the adhesiveness values measured using the Texture Analyzer, which highlighted an adhesiveness increase with GP addition in the dough (Table 1). Similar results have been reported by Shogren et al. [37] when evaluating the firmness perception in spaghetti fortified with soy flour. Moreover, no matter the GP amounts added, the panelist judged as acceptable the overall quality of the fortified pasta.

## 4. Conclusions

The results of this study showed that pasta fortification with GP caused an increase in the TPC and antioxidant activity in the cooked product. GP addition also affected the rate of starch digestibility, with a decrement of the RDS and an increment of the SDS, while the pGI, useful for predicting the likely in vivo glycemic response, showed a significant decrease with increasing amounts of GP in spaghetti. GP addition also influenced the pasta technological properties, increasing cooking loss, firmness, and adhesiveness in the cooked samples, while a decrease of the swelling index was observed. Furthermore, while pasta produced using only semolina had a fiber content of about 3 g fiber/100 g pasta, in the GP5- and GP10-fortified spaghetti, the fiber level ranged from 5.6 to 8.2 g fiber/100 g pasta, respectively. This is of the utmost interest because, according to the European Union, legislating a foodstuff as a “*source of fiber*” or “*high fiber*” is only possible if it contains at least 3 or 6 g of dietary fiber/100 g of product, respectively [38]. Moreover, the newly formulated pasta also showed good sensory acceptability. In conclusion, GP, a winemaking by-product available at low cost in great amounts, may represent an interesting ingredient to produce a functional pasta rich in antioxidant compounds and dietary fiber, with a potentially positive impact on the human health, also due to the assessed lower pGI in comparison with pure semolina spaghetti. While this study was focused on the use of GP obtained from a red grape variety rich in phenols, it would be of interest to carry out further studies to verify if the use of GP of different grape varieties may give the same positive results. Additionally, future studies should aim to assess the phenols bioaccessibility through in vitro digestion, as well as to characterize the different phenols moieties by using LC-MS analysis. The interaction between grape pomace and intestinal bacteria, as well as the healthy consequences, should be also investigated.

## Figures and Tables

**Table 1:** Cooking quality parameters and texture analysis of control pasta (GP0) and pasta fortified with different percentages of grape pomace (GP5 and GP10). The values are reported as mean ± standard deviation.

Pasta Samples	Optimum Cooking Time (min)	Cooking Loss (%)	Swelling Index (g Water/g Dry Pasta)	Firmness (N)	Adhesiveness (N)
GP0	6.0	6.61 ± 0.03 ^a^	3.59 ± 0.08 ^a^	104.20 ± 0.01 ^a^	−0.11 ± 0.01 ^a^
GP5	5.5	8.18 ± 0.23 ^b^	2.98 ± 0.21 ^b^	113.44 ± 0.02 ^b^	−0.45 ± 0.02 ^b^
GP10	5.0	9.48 ± 0.10 ^c^	0.97 ± 0.03 ^c^	135.80 ± 0.02 ^c^	−0.66 ± 0.02 ^c^

Values with different superscripts within the same column are significantly different for *p* < 0.05.

**Table 2:** Color analysis of uncooked and cooked control pasta (GP0) and pasta fortified with different percentages of grape pomace (GP5 and GP10). The data, reported as mean ± standard deviation, are expressed as L*, a*, and b* values.

Pasta Samples	L*		a*		b*	
	Uncooked	Cooked	Uncooked	Cooked	Uncooked	Cooked
GP0	65.95 ± 2.45 ^a^	70.13 ± 1.90 ^a^	−0.61 ± 0.34 ^b^	−5.57 ± 0.18 ^c^	16.18 ± 0.92 ^a^	14.24 ± 1.19 ^a^
GP5	47.64 ± 2.57 ^b^	43.28 ± 0.52 ^b^	1.46 ± 0.45 ^a^	4.84 ± 0.32 ^b^	6.50 ± 0.44 ^b^	6.06 ± 0.64 ^b^
GP10	43.55 ± 3.03 ^b^	33.43 ± 1.75 ^c^	1.62 ± 0.04 ^a^	6.61 ± 0.60 ^a^	5.27 ± 0.11 ^c^	5.27 ± 0.10 ^b^

Values with different superscripts within the same column are significantly different for *p* < 0.05.

**Table 3:** Total phenolic component (TPC) and antioxidant activity (FRAP and ABTS) of cooked and uncooked control pasta (GP0) and pasta fortified with different percentages of grape pomace (GP5 and GP10). The values are expressed as mean ± standard deviation.

Pasta Samples	TPC (mg GAE/g dw)	FRAP (µM TE/g dw)	ABTS (µM TE/g dw)
Uncooked GP0	0.43 ± 0.03 ^e^	0.30 ± 0.09 ^e^	2.85 ± 0.06 ^c^
Uncooked GP5	1.38 ± 0.04 ^c^	8.27 ± 0.27 ^c^	3.63 ± 0.18 ^b^
Uncooked GP10	2.57 ± 0.06 ^a^	11.32 ± 0.20 ^a^	4.50 ± 0.20 ^a^
Cooked GP0	0.15 ± 0.02 ^f^	0.09 ± 0.07 ^f^	0.60 ± 0.04 ^e^
Cooked GP5	1.05 ± 0.14 ^d^	7.34 ± 0.32 ^d^	2.46 ± 0.22 ^d^
Cooked GP10	1.81 ± 0.11 ^b^	9.42 ± 0.21 ^b^	4.40 ± 0.15 ^a^

Values with different superscripts within the same column are significantly different for *p* < 0.05.

**Table 4:** Starch fractions expressed as percentages of the total starch and predicted glycemic index (pGI) of cooked control pasta (GP0) and cooked pasta fortified with different percentages of grape pomace (GP5 and GP10). RDS: rapidly digested starch; SDS: slowly digested starch; RS: resistant starch. The values are reported as mean ± standard deviation.

Cooked Pasta Samples	Starch Fractions (%)			pGI
	RDS	SDS	RS	
GP0	33.45 ± 1.16 ^a^	32.94 ± 0.99 ^a^	2.13 ± 0.16 ^a^	57.46 ± 0.41 ^a^
GP5	31.87 ± 1.64 ^ab^	33.33 ± 1.36 ^a^	2.19 ± 0.17 ^a^	55.56 ± 0.22 ^b^
GP10	29.09 ± 2.05 ^b^	36.19 ± 0.34 ^b^	1.83 ± 0.41 ^a^	53.15 ± 1.37 ^c^

Values with different superscripts within the same column are significantly different for *p* < 0.05.
